# CaMKII Mediates Recruitment and Activation of the Deubiquitinase CYLD at the Postsynaptic Density

**DOI:** 10.1371/journal.pone.0091312

**Published:** 2014-03-10

**Authors:** Soe Thein, Jung-Hwa Tao-Cheng, Yan Li, K. Ulrich Bayer, Thomas S. Reese, Ayse Dosemeci

**Affiliations:** 1 Laboratory of Neurobiology, National Institute of Neurological Disorders and Stroke, National Institutes of Health, Bethesda, Maryland, United States of America; 2 EM Facility, National Institute of Neurological Disorders and Stroke, National Institutes of Health, Bethesda, Maryland, United States of America; 3 Protein/Peptide Sequencing Facility, National Institute of Neurological Disorders and Stroke, National Institutes of Health, Bethesda, Maryland, United States of America; 4 Department of Pharmacology, University of Colorado Denver School of Medicine, Aurora, Colorado, United States of America; Johannes Gutenberg University of Mainz, Germany

## Abstract

NMDA treatment of cultured hippocampal neurons causes recruitment of CYLD, as well as CaMKII, to the postsynaptic density (PSD), as shown by immunoelectron microscopy. Recruitment of CYLD, a deubiquitinase specific for K63-linked polyubiquitins, is blocked by pre-treatment with tatCN21, a CaMKII inhibitor, at a concentration that inhibits the translocation of CaMKII to the PSD. Furthermore, CaMKII co-immunoprecipitates with CYLD from solubilized PSD fractions, indicating an association between the proteins. Purified CaMKII phosphorylates CYLD on at least three residues (S-362, S-418, and S-772 on the human CYLD protein Q9NQC7-1) and promotes its deubiquitinase activity. Activation of CaMKII in isolated PSDs promotes phosphorylation of CYLD on the same residues and also enhances endogenous deubiquitinase activity specific for K63-linked polyubiquitins. Since K63-linked polyubiquitin conjugation to proteins inhibits their interaction with proteasomes, CaMKII-mediated recruitment and upregulation of CYLD is expected to remove K63-linked polyubiquitins and facilitate proteasomal degradation at the PSD.

## Introduction

Recruitment and activation of CaMKII produces several activity-induced changes at synapses [1]. CaMKII mediates these changes through a diverse set of downstream mechanisms, including, direct phosphorylation and upregulation of AMPA receptors [2], [3], anchoring of AMPA receptor to the postsynaptic density (PSD) via phosphorylation of TARPs, the adaptor proteins [4], [5], and regulation of the localization and activity of SynGAP through phosphorylation [6], [7]. Recent addition to this growing list is CaMKII-mediated recruitment and activation of proteasomes into spines [8], [9].

Ubiquitin-dependent proteasomal degradation has recently received a great deal of attention as a mechanism to modify the molecular composition of the PSD under excitatory conditions. Upon synaptic activity, the main PSD scaffolding molecules, PSD-95 [10], GKAP [11], [12], and Shank [12], as well as NMDA receptors [12], [13] are ubiquitinated and subsequently degraded through the ubiquitin-proteasome system.

Different types of ubiquitination determine different trafficking destinations for proteins. For example, while K48-linked polyubiquitination promotes proteasomal degradation [14], K63-linked polyubiquitination inhibits the interaction of a protein with proteasomes [15] and diverts it to non-proteasomal pathways, including but not limited to endocytosis [16], and autophagy [17]. The type and degree of protein ubiquitination are regulated by ubiquitin ligases that conjugate ubiquitins and deubiquitinases leading to their removal.

The PSD contains deubiquitinase activity for both K48-linked and K63-linked polyubiquitins [18]. Moreover, mass spectrometric analysis identifies CYLD, a deubiquitinase specific for K63-linked polyubiquitins [19], as one of the abundant proteins in the affinity-purified PSD fraction [20]. By immunoEM, CYLD has been shown to be present at the PSD and to accumulate further upon depolarization [18], suggesting a potential role of CYLD in activity-induced modification of PSD.

The present study examines the mechanism of activity-induced recruitment of CYLD to the PSD. We demonstrate that upon activation of NMDA receptors, CaMKII mediates recruitment and activation of CYLD at the PSD. Our results show that CYLD is a target of CaMKII and suggest a mechanism whereby CaMKII regulates deubiquitination and protein degradation at the PSD.

## Materials and Methods

Antibodies to CYLD: rabbit polyclonal (1∶250 for Western blots, 1∶100 for EM) is from Sigma (SAB4200060) and mouse monoclonal (E-4) used for immunoprecipitation is from Santa Cruz (sc-74434). Antibody to alphaCaMKII rabbit polyclonal (1∶100 for Western blots) is from Abcam (ab50202). Antibody to alphaCaMKII mouse monoclonal (6G9) (1∶100 for EM) is from Cayman Chemical (Ann Arbor, MI). Antibody to pan-ubiquitin: rabbit polyclonal (1∶200 or 1∶300 for Western blots) from Dako (Z0458).

Polyubiquitin chains are from Enzo Life Sciences (Farmingdale, NY). CYLD lysate (lysate from HEKT 293 cells overexpressing human CYLD transcript variant 2) is from Novus Biologicals (Littleton, CO). Purified recombinant human CYLD transcript variant 2 is from OriGene Technologies (Rockville, MD). N-methyl-D-aspartic acid (NMDA) and 2*R*-amino-5-phosphonovaleric acid (APV) are from Tocris (Ellisville, CO). Protease inhibitor cocktail (product # LD1273844) is from Thermo Scientific. KN-93 and autocamtide-2 –related inhibitory peptide (AIP) are from Calbiochem. For experiments with intact cells, the membrane-permeable CaMKII inhibitor peptide, tatCN21, was used. The inhibitor was made by fusing a cell-permeable “tat-sequence” with a 21-amino acid sequence (42–62) derived from the natural CaMKII inhibitor CaM-KIIN [21]. The control peptide is a scrambled peptide of the same 21-amino acid sequence fused to tat-sequence. For *in vitro* experiments with subcellular fractions and isolated proteins, the inhibitor peptide, CN21, without tat-sequence was used while the control peptide, 21-C, is another 21-amino acid sequence (48–68) from CaM-KIIN [22].

### Preparation and treatment of dissociated hippocampal cultures

The protocols for obtaining brains for hippocampal cultures were approved by the NIH Animal Use and Care Committee and conformed to NIH guidelines. Hippocampal cells from 21-day embryonic Sprague-Dawley rats were dissociated and grown on a glial cell layer as described by [23] for 3–4 weeks.

Control medium (124 mM NaCl, 2 mM KCl, 1.24 mM KH_2_PO_4_ 1.3 mM MgCl_2_, 2.5 mM CaCl_2_, 30 mM glucose in 25 mM HEPES at pH 7.4) was prepared, and where indicated, was supplemented to include 50 µM NMDA, 20 µM tatCN21, 20 µM tatcontrol. Cell cultures were washed once with control medium, and incubated for 20 min with or without peptides (tatCN21 or tatcontrol), followed by NMDA in the presence or absence of peptides for 2 minutes. Experiments using APV were carried out by washing cell cultures once with control medium, and subsequently incubating for 2 min with or without 50 µM NMDA either in the presence or absence of 50 µM APV. Treatment of samples was performed with dishes on a floating platform in a water bath at 37°C.

### Pre-embedding immunogold-labeling and electron microscopy

After treatment, neuronal cultures were processed for pre-embedding immunogold-labeling as described previously [24]. Briefly, cultures were fixed in 4% paraformaldehyde (EMS, Hatfield, PA) in PBS for 30–45 min at room temperature, permeabilized and blocked in 0.1% saponin and 5% normal goat serum for 40–60 min respectively, incubated with primary and secondary antibodies (Nanogold, Nanoprobes, Yaphank, NY) for 1–1.5 h, then fixed with 2% glutaraldehyde in PBS, silver enhanced (HQ kit, Nanoprobes), and processed for electron microscopy [24]. Only parallel samples from the same experiment were directly compared because the overall labeling sensitivity may differ between experiments.

### Morphometry and statistical analysis

Excitatory synapses are identified by clustered synaptic vesicles in the presynaptic terminal, the uniform 20 nm separation of the pre- and postsynaptic membrane, and the presence of a PSD, a layer of dense material underneath the postsynaptic membrane. The PSD complex was defined as the postsynaptic specialization that comprises the electron dense PSD core and a network contiguous to it. The area of the PSD complex to be measured was outlined by the postsynaptic membrane, a parallel dashed line drawn at 120 nm to the postsynaptic membrane, and two vertical lines to demarcate the area. The distance of the border from the postsynaptic membrane was set at 120 nm in order to avoid undercounting the label [25]. CYLD and CaMKII labels appear as individual black grains at the PSD complex, and immunolabeling intensity is expressed as the number of labels per µm length of the PSD. Only positively labeled PSDs (∼25–50% for CYLD [18] and >90% for CaMKII [24]) were included for measurement. One-way ANOVA followed by Tukey-posthoc test was performed to assess statistical significance of the differences between experimental groups with significance level set at *P*<0.05.

### Preparation of PSD fraction

PSD fraction from cerebral cortex was prepared as described previously [26] using brains from adult Sprague Dawley rats collected and frozen in liquid nitrogen within 2 min of decapitation by Pel-Freeze Biologicals (Rogers, AR).

### Endogenous phosphorylation at the PSD

PSD fractions were pre-incubated in 0.1 M DTT on ice for two hours. The PSD fractions (∼45 µg protein) were then incubated in phosphorylation medium in a final concentration of 1 mM CaCl_2_, and 40 µg/mL calmodulin (or 1 mM EGTA), 5 mM MgCl_2_, 50 µg/mL leupeptin, 20 mM DTT, 0.4 µM Microcystin-LR, with or without 100 µM ATP, in 20 mM HEPES, pH 7.4 in a final volume of 100 µL for 15 minutes at 37°C. When indicated, CaMKII inhibitor or control peptide was included at a final concentration of 2.5 µM (∼60 fold of the IC50) [27]. The samples were then divided into two equal volumes, one for Western immunoblotting to assess phosphorylation and another for deubiquitinase assays.

To examine the effect of phosphatase(s) on CYLD phosphorylation, PSD fractions (54 µg protein in a final volume of 90 µL) were incubated under phosphorylation conditions as described above, except that Microcystin-LR was excluded in one sample as indicated. The samples were subsequently centrifuged at 12,900 g for 10 min at 4°C, and the resulting supernatant was decanted to remove ATP. The pellets were resuspended in 80 µL of 20 mM HEPES, 1mM EGTA, with or without 0.4 µM Microcystin-LR and incubated at 37°C for one hour. The reaction was stopped by adding SDS-PAGE sample buffer.

### Phosphorylation of expressed CYLD using PSD fraction as source of kinase for mass spectrometry

PSD fractions (28 µg protein) pre-treated with 0.1 mM DTT were incubated with lysate (12 µg protein) from cells overexpressing human CYLD transcript variant 2 in phosphorylation medium in a final concentration of 1 mM CaCl_2_ and 40 µg/mL calmodulin (or 1 mM EGTA), 5 mM MgCl_2_, 20 mM DTT, 5% glycerol with or without 100 µM ATP, in 20 mM HEPES, pH 7.4 with protease and phosphatase inhibitor cocktails in a reaction volume of 28 µl for 15 minutes at 37°C. The reaction was stopped by adding SDS-PAGE sample buffer. The samples were resolved by SDS-PAGE and the gel band corresponding to CYLD was excised for mass-spectrometric analysis.

### Phosphorylation of purified CYLD with purified CaMKII for mass spectrometry

*In vitro* phosphorylation was performed by incubating 0.5 µg of purified CYLD with purified alphaCaMKII, at a final concentration of 10 nM, in medium containing1 mM CaCl_2_, 40 µg/mL calmodulin, 10 mM MgCl_2_, 0.025 mg/mL BSA, 4% glycerol, with or without 100 µM ATP in 20 mM HEPES, pH 7.4 in a final volume of 25 µl for 15 minutes at 37°C. The reaction was stopped by the addition of an equal volume of SDS-PAGE sample buffer. The samples were resolved by SDS-PAGE and the gel band corresponding to CYLD was excised for mass-spectrometric analysis.

### Mass-spectrometric analysis

Samples were alkylated with iodoacetamide, digested with trypsin overnight at 37°C. Peptides were analyzed by a nano-LC/MS/MS system with an Ultimate 3000 HPLC (Thermo-Dionex) connected to an Orbitrap Elite mass spectrometer (Thermo Scientific) via an Easy-Spray ion source (Thermo Scientific). Peptides were separated on ES800 Easy-Spray column (75 µm inner diameter, 15 cm length, 3 µm C18 beads; Thermo Scientific) at a flow rate of 300 nl/min with a 25 min linear gradient of 2–27% mobile phase B (mobile phase A: 2% acetonitrile, 0.1% formic acid; mobile phase B: 98% acetonitrile, 0.1% formic acid). Thermo Scientific Orbitrap Elite mass spectrometer was operated in positive nano-electrospray mode. MS data were acquired in both profile and data dependent modes. The resolution of the survey scan was set at 60 k at m/z 400 with a target value of 1×10^6^ ions. The m/z range for MS scans was 300–1600. The isolation window for MS/MS fragmentation was set to 1.9 and the top two most abundant ions were selected for product ion analysis. Ion trap enhanced scan rate was used for the MS/MS data acquisition with decision tree procedure activated. Dynamic exclusion was 9 s and early expiration was abled. The Xcalibur RAW files were converted to peak list files in mgf format using Mascot Distiller (version 2.4.3.3). Database search was performed using Mascot Daemon (2.4.0) against the NCBI human database.

### Deubiquitinating assays

#### Endogenous deubiquitinase activity at the PSD

The endogenous phosphorylation reaction with the PSD fraction was carried out as described above and 50 µl aliquots were taken for deubiquitinase assay. An equal volume of solution containing 10 mM EGTA, 10 mM EDTA, and phosphatase inhibitor cocktail in 100 mM HEPES pH 7.5 was added to the reaction mixture. Aliquots of 20 µl (∼4.5 µg of PSD proteins) were incubated with 5 µL of either K63- or K48-linked polyubiquitin chains (total 1 µg in 0.5 mg/mL BSA) at 37°C for indicated time intervals. Reactions were terminated by adding SDS-PAGE sample buffer for Western analysis.

#### Effect of CaMKII-mediated phosphorylation on deubiquitinase activity of purified CYLD

*In vitro* phosphorylation was performed by incubating 1.5 µg of purified CYLD with purified alphaCaMKII, at a final concentration of 15 nM, in medium containing 1 mM CaCl_2_, 40 µg/mL calmodulin, 10 mM MgCl_2_, 0.02 mg/mL BSA, 16% glycerol, 1 mM DTT, 50 µg/µL leupeptin and phosphatase inhibitor cocktail in 20 mM HEPES, pH 7.4, with or without 100 µM ATP in a final volume of 32 µL for 15 minutes at 37°C. An equal volume of a solution containing 10 mM EGTA, 10 mM EDTA, 10 mM DTT in 100 mM HEPES pH 7.5 was added to the reaction. Subsequently, 15 µL aliquots containing ∼0.36 µg of CYLD were incubated with 2.5 µL of K63-linked polyubiquitin chains (0.5 µg total in 0.5 mg/ml BSA) at 37°C for indicated time intervals. The heat-inactivated control consisted of boiling the sample for two minutes prior to incubation with K63-linked polyubiquitins for one hour. Reactions were terminated by adding SDS-PAGE buffer for Western analysis.

### Electrophoresis and immunoblotting

Samples were resolved by SDS-PAGE on either 7.5% Mini-PROTEAN TGX gels from BioRAD or 4–12% gradient Bis-Tris gels from Life Technologies, and transferred to PVDF membranes, blocked, incubated with specified primary antibodies and then with horseradish peroxidase-conjugated secondary antibodies (1∶50,000 dilution), and the signal was finally visualized by chemiluminescence (SuperSignal West Pico, Thermo Scientific).

### Immunoprecipitation from solubilized PSD fractions

The PSD fractions (150 µg protein) were solubilized in 1% SDS in a final volume of 88 µL in 20 mM HEPES, pH 7.4 by continuous vortexing for three minutes. The sample was then diluted with TX-100 to 1 mL final volume to make up a final concentration of 0.5% TX-100, supplemented with protease inhibitor cocktail and centrifuged at 4°C, 20,800 g for 45 minutes. The supernatant was divided into two equal volumes, and 50 µL of 2% TX-100 was added to both. 50 µL (10 µg) of CYLD antibody was added to the first, but not to the second control tube. The samples were incubated for 2 hours at room temperature on an end-over-end rotator. Then, the samples were incubated with protein-A conjugated agarose beads (Thermo Scientific, product # 22811, 400 µL initial slurry washed three times with PBS) for 2 hours at room temperature or overnight 4°C on the rotator. The beads were washed six times with 1% TX-100 in PBS and then rinsed once with PBS. The immunoprecipitate was eluted with electrophoresis sample buffer and stored at −20°C until Western blot analysis.

## Results

### Accumulation of CYLD at the PSD is promoted by NMDA and blocked by tatCN21

**Figure 1:**
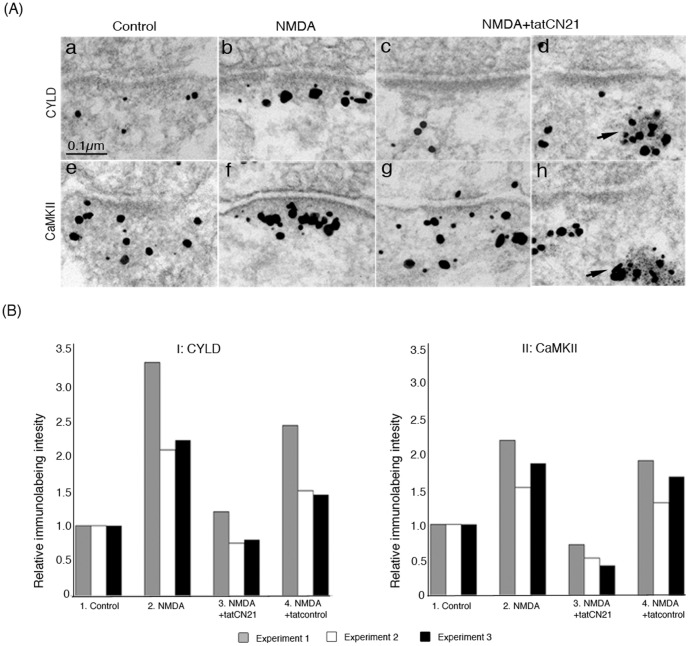
Recruitment of CYLD to the PSD is dependent on translocation of CaMKII to the PSD. Hippocampal cultures were pre-incubated for 20 min with or without the CaMKII inhibitor tatCN21 followed by the application of NMDA for 2 min. Parallel controls were incubated in medium alone. (**A**) Electron micrographs depicting immunogold labeling (dark grains of heterogeneous sizes) for CYLD (upper panel) or CaMKII (lower panel) at the synaptic region. Both CYLD (b) and CaMKII (f) accumulate at the PSD upon NMDA-treatment. Addition of tatCN21 blocks the NMDA-induced accumulation of both CYLD and CaMKII at the PSD. TatCN21-induced CaMKII-polyribosome aggregates (arrow in h) also label for CYLD (arrow in d). (**B**) Measurements of immunolabeling intensity for CYLD (I) and CaMKII (II) at the PSD in response to different treatments. Data were collected from three experiments, where sister cultures were labeled in parallel for CYLD and CaMKII. Mean immunolabeling intensities at the PSD (number of gold particles per unit length of PSD) were normalized with respect to the control group. Statistical analysis was carried out with one-way ANOVA followed by Tukey-posthoc test for individual experiments with significance level set at *P*<0.05. For CYLD (BI): 1 vs. 2, significant in all three experiments; 2 vs. 3, significant in all three experiments; 1 vs. 3, not significant in all three experiments, 2 vs. 4, not significant in two out of three experiments; 3 vs. 4, significant two out of three experiments. For CaMKII (BII): 1 vs. 2, significant in all three experiments; 2 vs. 3, significant in all three experiments; 1 vs. 3, significant in two out of three experiments; 2 vs. 4, not significant in all three experiments; 3 vs. 4, significant in all three experiments.

Conditions for recruitment of CYLD at the PSD were investigated by immunogold electron microscopy on dissociated hippocampal cultures. Treatment with 50 µM NMDA for 2 min resulted in the accumulation of CYLD (Fig. 1 A, top panel, b vs. a) and CaMKII (Fig. 1A, lower panel, f vs. e) at the PSD. The NMDA-induced accumulation of CYLD as well as CaMKII at the PSD was blocked by the NMDAR antagonist APV (Table S1). These results suggest that similar to CaMKII [28], CYLD is recruited to the PSD in response to NMDA receptor activation.

Because the two proteins exhibit similar redistribution pattern in response to NMDA, we then tested whether CaMKII is involved in NMDA-induced recruitment of CYLD. TatCN21, a cell-penetrating CaMKII inhibitor, at 20 µM blocked both CaMKII and CYLD accumulation at the PSD induced by NMDA (Fig. 1 A, c vs. b for CYLD and g vs. f for CaMKII, respectively).

CYLD and CaMKII immunolabeling intensities at the PSD under different incubation conditions were quantified (Fig. 1B). Data were collected from three experiments where sister cultures were labeled in parallel for CYLD and CaMKII. Labeling intensities for CYLD and CaMKII at the PSD increased significantly, ∼2.5 fold and ∼1.8 fold respectively, upon NMDA treatment. When tatCN21 was applied prior to NMDA, the labeling intensities for CaMKII and CYLD at the PSD were reduced to 30% and 36% respectively of the values in samples treated with NMDA only. The effect of the control peptide (tatcontrol), on the other hand, was much smaller, reducing labeling intensities for CaMKII and CYLD to 88% and 70% respectively. These results indicate that inhibition of CaMKII translocation via tatCN21 blocks CYLD translocation as well.

Additionally, tatCN21 at 20 µM induced formation of CaMKII-polyribosome aggregates in neuronal cultures. TatCN21-induced CaMKII-polyribosome aggregates often appeared near synapses (Fig. 1 A h). Under the same conditions, these morphologically recognizable aggregates also labeled for CYLD (Fig. 1 A d). CaMKII-polyribosome aggregates that form throughout the dendritic shaft also consistently labeled for CYLD (data not shown). These results collectively indicate that the re-distribution of CYLD in response to NMDA and tatCN21mirrors that of CaMKII and suggests an association between the two proteins.

### CaMKII co-immunoprecipitates with CYLD and promotes phosphorylation of CYLD at the PSD

**Figure 2:**
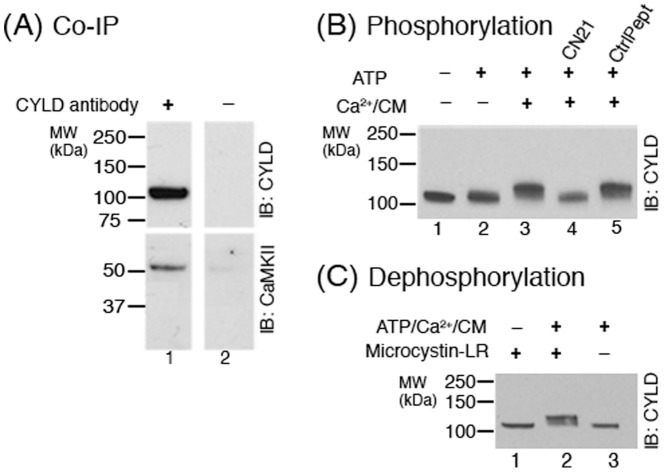
CaMKII co-immunoprecipitates with CYLD and promotes phosphorylation of CYLD at the PSD. (**A**) CYLD in solubilized PSD fractions was immunoprecipitated using CYLD antibody, followed by Western immunoblotting with antibodies for either CYLD or CaMKII. CaMKII co-immunoprecipitated with CYLD while neither protein was detected when beads without CYLD antibody were used. Two independent experiments yielded similar results. (**B**) PSD fractions were incubated under various conditions designed to manipulate CaMKII activity, followed by Western immunoblotting with CYLD antibody. Addition of Ca^2+^/calmodulin along with ATP caused a distinct mobility shift in CYLD (lane 3), indicative of phosphorylation. The mobility shift was prevented upon addition of CN21, a CaMKII inhibitor (lane 4). The control peptide had no appreciable effect on the observed mobility shift (lane 5). Two independent experiments yielded similar results. (**C**) PSD fractions were incubated under conditions designed to manipulate endogenous kinases and phosphatases at the PSD, followed by Western immunoblotting with CYLD antibody. The mobility shift elicited by ATP/Ca^2+^/CM was reversed upon incubation in the absence of the phosphatase inhibitor MicrocystinLR (lane 3).

Potential association between CYLD and CaMKII was examined by immunoprecipitation. PSD fractions were solubilized with SDS, diluted in Triton X-100 and then immunoprecipitated with CYLD antibody. Western immunoblots of the immunoprecipitates showed that CaMKII co-immunoprecipitated with CYLD (Fig. 2A).

These results led us to further examine whether CaMKII promotes phosphorylation of CYLD at the PSD. PSD fractions were incubated under different conditions designed to manipulate CaMKII activity. Phosphorylation of CYLD was assessed through changes in its electrophoretic mobility in Western Immunoblots. Addition of Ca^2+^/calmodulin along with ATP caused a shift in the mobility of the CYLD band (Fig. 2B, lane 3), which was reversed upon inclusion of the CaMKII inhibitor peptide, CN21 (Fig. 2B, lane 4). In contrast to other CaMKII inhibitors, CN21 is highly selective for CaMKII and does not affect other CaM kinases, such as CaMKI, CaMKIV, or DAPK1 [22]. The control peptide had no appreciable effect on the Ca^2+^/calmodulin-dependent phosphorylation of CYLD (Fig. 2B, lane 5). Two additional CaMKII inhibitors tested, KN-93 and AIP, also showed an inhibitory effect, although, unlike CN21, the reversal of the mobility shift was only partial (Fig. S1). Together, these data indicate that CaMKII activation promotes phosphorylation of CYLD at the PSD.

The mobility change in CYLD, elicited by the incubation of the PSD fraction in the presence of Ca^2+^/calmodulin and ATP, could not be maintained in the absence of the phosphatase inhibitor MicrocystinLR (Fig. 2C). This observation indicates that CYLD can be dephosphorylated by endogenous type1 and/or type 2A phosphatase(s) at the PSD.

### CaMKII phosphorylates CYLD on multiple residues

Two *in vitro* phosphorylation protocols were used to identify CaMKII-mediated phosphorylation sites on CYLD and test whether CaMKII phosphorylates CYLD directly. In the first protocol, lysate from HEK293 cells overexpressing human CYLD was incubated with PSD fractions under three different media containing 1) neither ATP nor Ca^2+^/calmodulin, 2) ATP only, or 3) both ATP and Ca^2+^/calmodulin. Upon addition of both ATP and Ca^2+^/calmodulin, three phosphorylated peptides (SELFYTLNGSS**p**VDSQPQSK, FHS**p**LPFSLTK, IFPS**p**LELNITDLLEDTPR), with phosphorylated serine residues denoted as S**p**, were detected by LC/MS/MS analysis (Table 1). The corresponding positions of these phosphorylated serine residues on the human CYLD isoform 1 (Q9NQC7-1) were S-362, S-418, and S-772. One of these phosphorylated peptides (FHS**p**LPFSLTK) was also detected in the presence of ATP only.

**Table 1:** Phosphorylated peptides of CYLD detected under CaMKII activating conditions.

Phosphorylated Sequence	CYLD+PSD	CYLD+CaMKII
SELFYTLNGSS**p**VDSQPQSK (S-362)	1/3 experiments*	2/2 experiments
FHS**p**LPFSLTK (S-418)	3/3 experiments	1/2 experiments
IFPS**p**LELNITDLLEDTPR (S-772)	2/3 experiments	1/2 experiments

Recombinant human CYLD was incubated with either PSD fraction (CYLD+PSD) or purified CaMKII (CYLD+CaMKII) and analyzed by LC/MS/MS to identify phosphorylated residues. Sequences above were detected only when samples were incubated in the presence of ATP and Ca^2+^/calmodulin, except for FHS**p**LPFSLTK (S-418), which was also detected when CYLD was incubated with PSDs in the presence of ATP only. Number of experiments where a phosphorylated sequence was detected/total number of experiments is shown. The phosphorylated residues are indicated as S**p**, and the corresponding positions on human CYLD isoform 1 (Q9NQC7-1) are indicated in parenthesis next to the sequence.

*LC/MS/MS analysis failed to differentiate between S-361 and S-362 as a phosphorylated residue.

To confirm that CaMKII phosphorylates CYLD at the PSD directly rather than via sequential phosphorylation through activation of other kinases, phosphorylation of purified CYLD by purified CaMKII was tested. The two proteins were incubated in Ca^2+^/calmodulin-containing medium in the presence or absence of ATP. Addition of ATP led to phosphorylation of the same three residues, indicating that CaMKII directly phosphorylates CYLD on these residues. Consistent with mass-spectrometric results, Western immunoblots using an antibody specific for phospho CYLD (S-418) showed that purified CaMKII phosphorylates purified CYLD on this residue (Fig. S2- left). However, in the PSD fraction, CaMKII inhibitor CN21 could not completely block the phosphorylation of CYLD at S-418, implicating involvement of a second kinase in its phosphorylation (Fig. S2- right).

One additional phosphopeptide, GVGDKG[SSS]**p**HNKPK was detected only in one experiment using the first protocol, where lysate from cells overexpressing CYLD was incubated with PSD fraction in the presence of ATP and Ca^2+^/calmodulin. However, it was not detected in the experiments using purified proteins. Thus, CaMKII directly phosphorylates at least three residues on CYLD, while the identity of the kinase responsible for phosphorylating the additional fourth residue is unclear.

### CaMKII-mediated phosphorylation enhances CYLD activity

**Figure 3:**
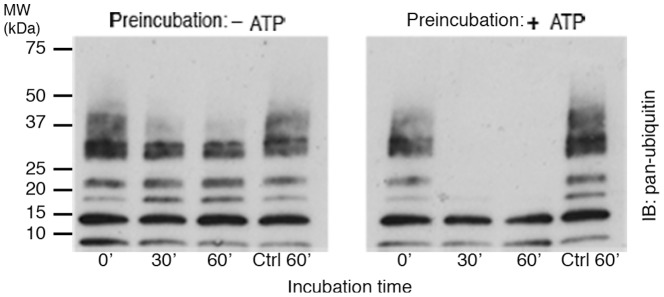
CaMKII-mediated phosphorylation upregulates CYLD deubiquitinase activity. Purified CYLD was pre-incubated with purified CaMKII in Ca^2+^/calmodulin-containing medium in the presence or absence of ATP. The samples were subsequently incubated with K63-linked polyubiquitins for different times, followed by Western immunoblotting with a pan-ubiquitin antibody. The rate of degradation of K63-linked polyubiquitins increased when CYLD was pre-incubated with CaMKII in ATP-containing medium. Parallel controls (Ctrl 60′) were heat-inactivated prior to incubation with K63-linked polyubiquitins for 60 minutes. Two experiments yielded similar results.

Having established that CaMKII phosphorylates CYLD, we further tested the effect of CaMKII-mediated phosphorylation on its activity. Purified CYLD was pre-incubated with purified CaMKII in medium containing Ca^2+^/calmodulin in the absence or presence of ATP, and subsequently incubated with K63-linked polyubiquitin chains for different time intervals (0′, 30′, 60′). Changes in activity were assessed by comparing the level of degradation of polyubiquitins at each time point in Western blots using an antibody for pan-ubiquitin. Activation of CaMKII by the addition of ATP promotes activation of CYLD, as indicated by the increase in the rate of degradation of polyubiquitins (Fig. 3). These results with purified proteins show that CaMKII activates CYLD via phosphorylation.

### CaMKII promotes deubiquitinase activity specific for K63-linked polyubiquitins at the PSD

**Figure 4:**
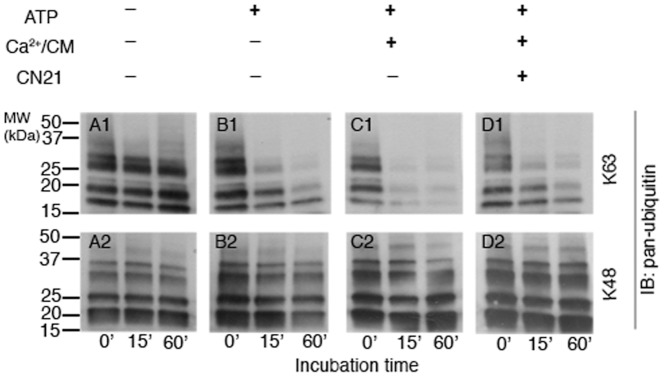
Activation of CaMKII upregulates deubiquitinase activity at the PSD. PSD fractions were treated under four different conditions designed to manipulate CaMKII activity as indicated above. The samples were subsequently incubated with either K63- or K48-linked polyubiquitins for different time intervals, followed by Western immunoblotting with a pan-ubiquitin antibody. The rate of degradation of K63-linked polyubiquitins increased when the PSD was pre-incubated with ATP, Ca^2+^/calmodulin, conditions that promote CaMKII activation (C1). Inclusion of CN21, a CaMKII inhibitor, prevented Ca^2+^/calmodulin-dependent upregulation of deubiquitinase activity (D1). On the other hand the rate of degradation of K48-linked polyubiquitins did not show appreciable change in correlation with CaMKII activity (C2). Two independent experiments showed similar results.

Potential involvement of CaMKII in regulating deubiquitinase activity at the PSD was further examined. PSD fractions were pre-treated under various conditions designed to manipulate CaMKII activity, and were subsequently incubated with either K63- or K48-linked polyubiquitin chains of varying lengths for different time intervals (0′, 15′, 60′). Changes in deubiquitinase activity were assessed by comparing the degradation of polyubiquitin chains at each time point in Western blots. Preincubation with Ca^2+^/calmodulin together with ATP increases the rate of degradation of K63-linked polyubiquitins (Fig. 4C1). The Ca^2+^/calmodulin-dependent upregulation of deubiquitinase activity for K63-linked polyubiquitins was blocked by the CaMKII inhibitor CN21 (Fig. 4D1) while a control peptide had no appreciable effect (data not shown). These results indicate that CaMKII activation promotes deubiquitinase activity at the PSD for K63-linked polyubiquitins. In contrast, the rate of degradation of K48-linked polyubiquitins did not show any appreciable change in response to CaMKII activation (Fig. 4C2). Furthermore, addition of ATP in the absence of Ca^2+^/calmodulin promoted a relatively modest increase in the rate of K63-linked polyubiquitin breakdown (Fig. 4B1), suggesting that an endogenous Ca^2+^-independent kinase activity also regulates deubiquitinase activity specific for K63-linked polyubiquitins at the PSD.

## Discussion

NMDA receptor activation promotes accumulation of both CaMKII and CYLD at PSDs in hippocampal neurons, as demonstrated by immunogold electron microscopy. TatCN21, a CaMKII inhibitor, inhibits the translocation of CaMKII at the PSD induced by NMDA [24]. We now demonstrate that accumulation of CYLD at the PSD induced by NMDA is also blocked by tatCN21, indicating that recruitment of CYLD to the PSD may depend on translocation of CaMKII.

TatCN21 induces formation of CaMKII-polyribosome complexes, which also label for CYLD. That CYLD colocalizes with CaMKII at different locations under different incubation conditions suggests a physical association of the two proteins. Co-immunoprecipitation of the two proteins from solubilized PSD fractions confirmed an association between CYLD and CaMKII. Similarly, the delta-subunit of CaMKII was previously shown to co-immunoprecipitate with CYLD from HEK293T cells extracts [29]. Altogether biochemical and electron microscopic evidence suggests that in response to NMDA receptor activation, CYLD is recruited to the PSD through its association with CaMKII. The association between CYLD and CaMKII may not require CaMKII kinase activity because CYLD appears to colocalize with CaMKII in tatCN21-induced polyribosome aggregates, despite the inhibition of CaMKII activity by tatCN21.

Although CaMKII and CYLD co-immunoprecipitate from solubilized PSDs, the association between CYLD and CaMKII may require additional factors such as posttranslational modifications or adaptor proteins. In fact, even though there is a large excess of CaMKII in the PSD fraction compared to CYLD, addition of recombinant CYLD to the solubilized PSDs did not pull down more CaMKII using a CYLD antibody (data not shown). This suggests a lack of binding between recombinant CYLD and PSD-associated CaMKII. On the other hand, consistent with our electron microscopy data, CaMKII kinase activity is probably not required for the association between the two molecules because CaMKII co-immunoprecipitates with CYLD under non-phosphorylating conditions.

While CaMKII enzymatic activity does not appear to be necessary for CYLD recruitment to the PSD, CaMKII nonetheless can phosphorylate CYLD. Activation of endogenous CaMKII in isolated PSDs promotes phosphorylation of CYLD. CYLD phosphorylation can be reversed by endogenous phosphatase activity, indicating another layer of regulation of CYLD phosphorylation at the PSD.

Incubation of the recombinant CYLD with PSD fractions in the presence of Ca^2+^/calmodulin and ATP leads to phosphorylation on at least three serine residues, corresponding to S-362, S-418 and S-772 on human CYLD isoform 1 (Q9NQC7-1). Incubating purified CYLD and purified CaMKII promotes phosphorylation on the same serine residues, providing evidence that the phosphorylation of CYLD in the PSD fractions is directly mediated by the endogenous CaMKII.

Mass spectrometric analysis on the recombinant CYLD incubated with PSD fractions showed that addition of ATP can induce phosphorylation at S-418, even in the absence of Ca^2+^, a result confirmed by Western immunoblotting experiments with an antibody specific for phospho CYLD (S-418) (Fig. S2). Western immunoblotting experiments further demonstrated phosphorylation of this residue at the PSD even in the presence of CN21 (Fig. S2). Together, these observations indicate that, in addition to CaMKII, a Ca^2+^-independent kinase at the PSD phosphorylates CYLD at S-418.

CaMKII-mediated phosphorylation of CYLD enhances its deubiquitinase activity as shown in *in vitro* experiments using purified proteins. Similarly, activation of CaMKII in isolated PSDs leads to a significant increase in endogenous deubiquitinase activity targeting K63-linked polyubiquitins. Since CYLD is a major protein in isolated PSDs [20] and is phosphorylated by CaMKII, it is presumed that the observed changes in deubiquitination are a result of CaMKII-mediated activation of CYLD.

Among the three sites on CYLD phosphorylated by CaMKII, S-418 has been reported to be phosphorylated by IKK, resulting in an inhibition of CYLD activity [30], [31]. A possibility to reconcile these apparently contradictory results could be that phosphorylation of additional sites, S-362 and S-772, by CaMKII overrides the inhibitory effect of S-418 phosphorylation and promotes activation instead. Indeed, a similar scenario where phosphorylation on one residue overrides the effect of phosphorylation on another residue has been described in the regulation glycogen synthase kinase 3β [32].

Collectively, our results imply that following activation of NMDA receptors, CaMKII upregulates the removal of K63-linked polyubiquitins by recruiting and activating CYLD at the PSD. Because conjugation of K63-linked polyubiquitins to proteins prevents their association with the proteasomes [15], CaMKII-mediated recruitment and activation of CYLD should facilitate proteasomal degradation of PSD proteins. Thus, regulation of CYLD by CaMKII can function as a switch that channels trafficking of proteins, previously flagged for alternative pathways, into proteasomal degradation.

Recruitment and activation of CYLD at the PSD appears to be complementary to CaMKII-mediated synaptic recruitment and activation of the proteasome, described by Djakovic et al. and Bingol et al. [8], [9_ENREF_9]. The concurrent recruitment and activation of CYLD and of proteasomes mediated by CaMKII may coordinate to modify local protein degradation. Once recruited and activated at the PSD, CYLD would facilitate the association between PSD proteins and proteasomes by removing K63-linked polyubiquitins, allowing proteasomes to degrade the proteins. Thus, emerging evidence points out that by targeting both proteasomes and CYLD, CaMKII can function as a central regulator of proteasomal degradation at the PSD.

## Supporting Information

Figure S1 **Effect of KN93 and AIP on Ca^2+^/calmodulin-dependent phosphorylation of CYLD.** PSD fractions were incubated under different conditions designed to manipulate CaMKII activity, followed by Western immunoblotting with CYLD antibody. Addition of Ca^2+^/calmodulin along with ATP caused a shift in mobility of CYLD, indicative of phosphorylation. The observed mobility shift was partially reversed upon inclusion of CaMKII inhibitors, 40 µM KN-93 or 20 µM AIP.
(TIF)

Figure S2 **Phosphorylation of CYLD at S-418 assessed with a phospho-specific antibody.** (**Left**) Purified CaMKII was incubated with purified CYLD in Ca^2+^/calmodulin-containing medium in the presence or absence of ATP, followed by Western immunoblotting. Addition of ATP induced the appearance of a band recognized by an antibody specific for CYLD phosphorylated at S-418. (**Right**) PSD fractions were incubated under different conditions designed to manipulate CaMKII activity, followed by Western immunoblotting with phospho-CYLD (S-418) specific antibody. Addition of ATP alone induced phosphorylation of CYLD at S-418 (lane 2). Inclusion of Ca^2+^/calmodulin together with ATP caused a mobility shift of the band corresponding to phospho-CYLD (lane 3), suggesting phosphorylation of additional residues. The observed Ca^2+^/calmodulin-dependent mobility shift was blocked upon inclusion of the CaMKII inhibitor CN21 (lane 4) but phosphorylation at Ser-418 was, at least partially, preserved, suggesting phosphorylation of S-418 by additional kinases(s) present at the PSD. Two independent experiments yielded similar results.
(TIF)

Table S1 **Percent label intensity at the PSD compared to controls.** In one EM experiment, sister hippocampal cultures were treated with either NMDA alone or NMDA plus APV (50 µM) for 2 min and labeled for either CYLD or CaMKII, followed by the quantification of immubolabeling intensities at the PSD. The differences between NMDA and NMDA+APV groups were statistically significant for both proteins (*P*<0.005, ANOVA with Tukey post-test).
(DOCX)
