# Correction: CaMKII Mediates Recruitment and Activation of the Deubiquitinase CYLD at the Postsynaptic Density

**DOI:** 10.1371/journal.pone.0095901

**Published:** 2014-04-16

**Authors:** The PLOS ONE Staff

The authors would like to provide higher-quality images for Figures 2, 3, and 4. Please view these figures here.

**Figure 2:**
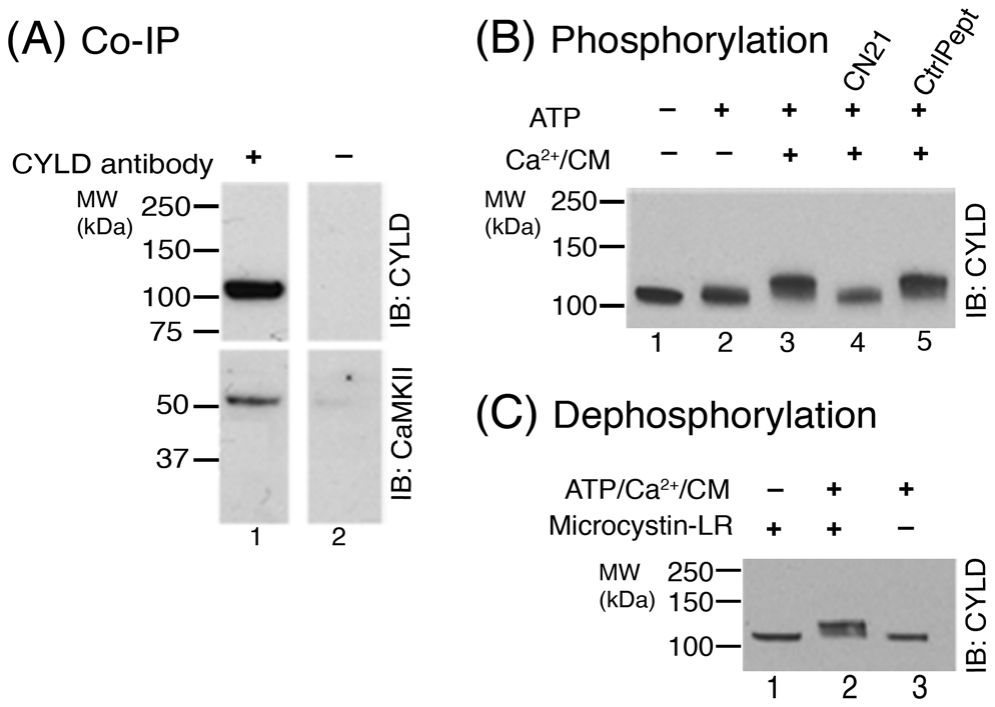
CaMKII co-immunoprecipitates with CYLD and promotes phosphorylation of CYLD at the PSD. (**A**) CYLD in solubilized PSD fractions was immunoprecipitated using CYLD antibody, followed by Western immunoblotting with antibodies for either CYLD or CaMKII. CaMKII co-immunoprecipitated with CYLD while neither protein was detected when beads without CYLD antibody were used. Two independent experiments yielded similar results. (**B**) PSD fractions were incubated under various conditions designed to manipulate CaMKII activity, followed by Western immunoblotting with CYLD antibody. Addition of Ca^2+^/calmodulin along with ATP caused a distinct mobility shift in CYLD (lane 3), indicative of phosphorylation. The mobility shift was prevented upon addition of CN21, a CaMKII inhibitor (lane 4). The control peptide had no appreciable effect on the observed mobility shift (lane 5). Two independent experiments yielded similar results. (**C**) PSD fractions were incubated under conditions designed to manipulate endogenous kinases and phosphatases at the PSD, followed by Western immunoblotting with CYLD antibody. The mobility shift elicited by ATP/Ca^2+^/CM was reversed upon incubation in the absence of the phosphatase inhibitor MicrocystinLR (lane 3).

**Figure 3:**
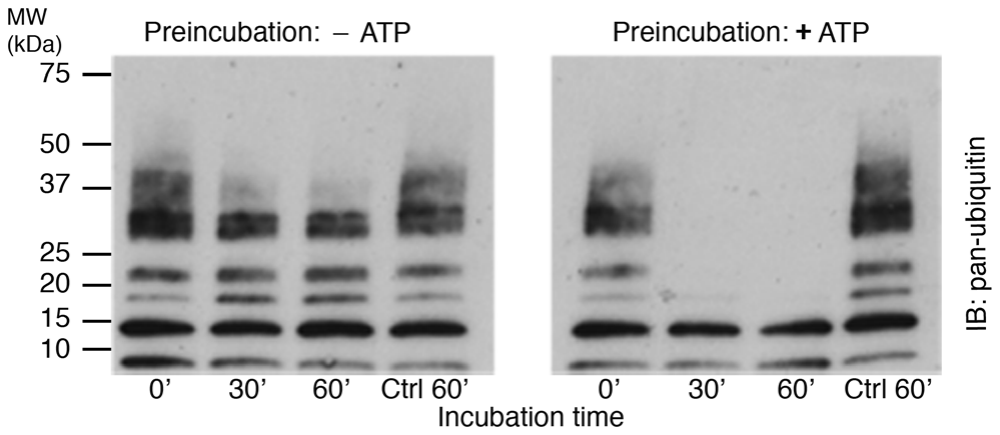
CaMKII-mediated phosphorylation upregulates CYLD deubiquitinase activity. Purified CYLD was pre-incubated with purified CaMKII in Ca^2+^/calmodulin-containing medium in the presence or absence of ATP. The samples were subsequently incubated with K63-linked polyubiquitins for different times, followed by Western immunoblotting with a pan-ubiquitin antibody. The rate of degradation of K63-linked polyubiquitins increased when CYLD was pre-incubated with CaMKII in ATP-containing medium. Parallel controls (Ctrl 60′) were heat-inactivated prior to incubation with K63-linked polyubiquitins for 60 minutes. Two experiments yielded similar results.

**Figure 4:**
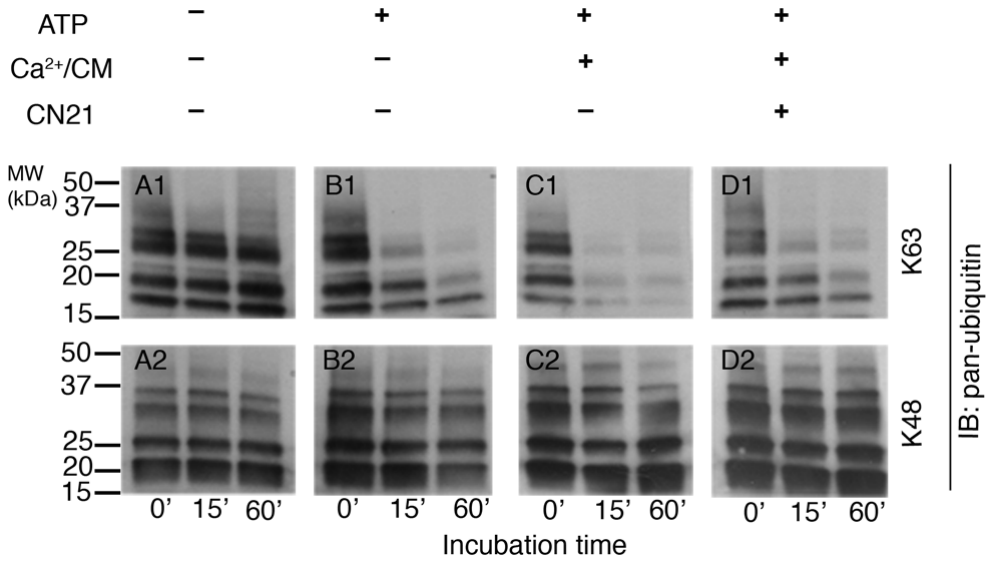
Activation of CaMKII upregulates deubiquitinase activity at the PSD. PSD fractions were treated under four different conditions designed to manipulate CaMKII activity as indicated above. The samples were subsequently incubated with either K63- or K48-linked polyubiquitins for different time intervals, followed by Western immunoblotting with a pan-ubiquitin antibody. The rate of degradation of K63-linked polyubiquitins increased when the PSD was pre-incubated with ATP, Ca^2+^/calmodulin, conditions that promote CaMKII activation (C1). Inclusion of CN21, a CaMKII inhibitor, prevented Ca^2+^/calmodulin-dependent upregulation of deubiquitinase activity (D1). On the other hand the rate of degradation of K48-linked polyubiquitins did not show appreciable change in correlation with CaMKII activity (C2). Two independent experiments showed similar results.
