# Supplementary figures and images for: CaMKII Mediates Recruitment and Activation of the Deubiquitinase CYLD at the Postsynaptic Density
Source: PLoS One. 2014 Mar 10;9(3):e91312. doi: 10.1371/journal.pone.0091312 (PMC3948843; doi:10.1371/journal.pone.0091312)

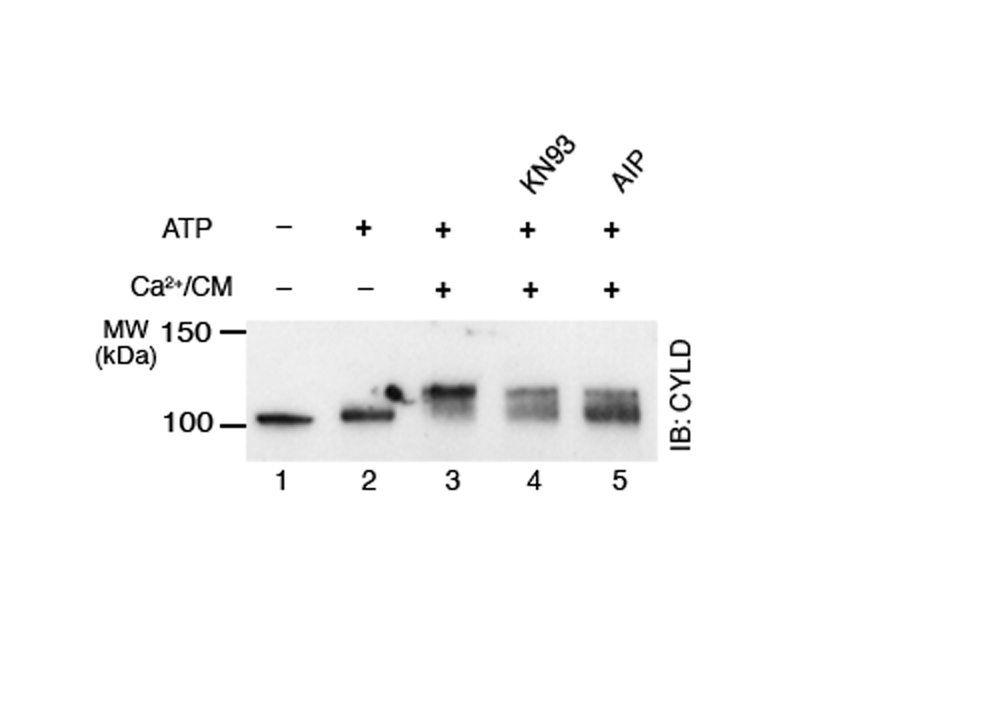

Supplement: Figure S1 — Effect of KN93 and AIP on Ca2+/calmodulin-dependent phosphorylation of CYLD. PSD fractions were incubated under different conditions designed to manipulate CaMKII activity, followed by Western immunoblotting with CYLD antibody. Addition of Ca2+/calmodulin along with ATP caused a shift in mobility of CYLD, indicative of phosphorylation. The observed mobility shift was partially reversed upon inclusion of CaMKII inhibitors, 40 µM KN-93 or 20 µM AIP. (TIF) [file pone.0091312.s001.tif]

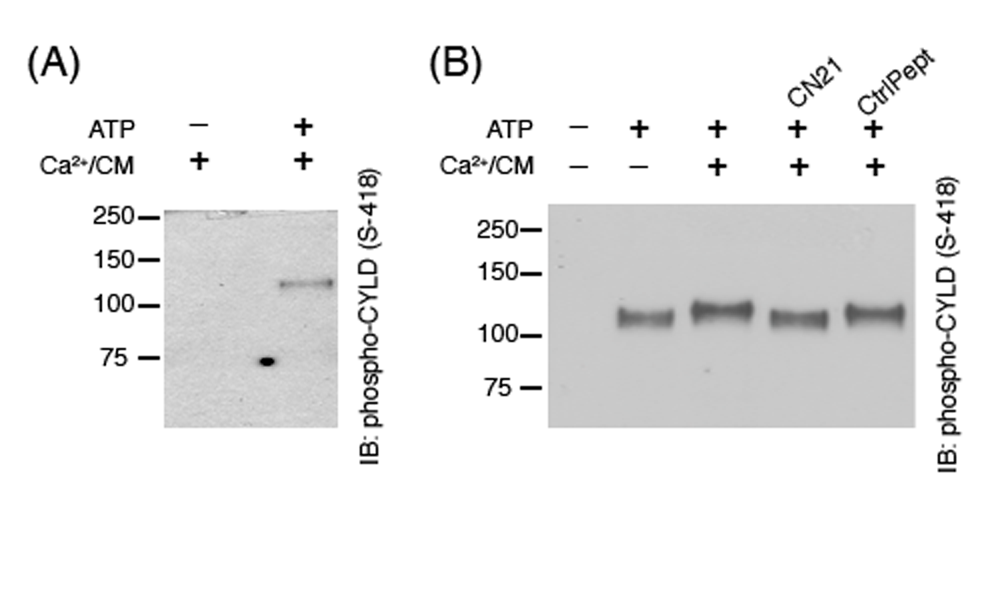

Supplement: Figure S2 — Phosphorylation of CYLD at S-418 assessed with a phospho-specific antibody. (Left) Purified CaMKII was incubated with purified CYLD in Ca2+/calmodulin-containing medium in the presence or absence of ATP, followed by Western immunoblotting. Addition of ATP induced the appearance of a band recognized by an antibody specific for CYLD phosphorylated at S-418. (Right) PSD fractions were incubated under different conditions designed to manipulate CaMKII activity, followed by Western immunoblotting with phospho-CYLD (S-418) specific antibody. Addition of ATP alone induced phosphorylation of CYLD at S-418 (lane 2). Inclusion of Ca2+/calmodulin together with ATP caused a mobility shift of the band corresponding to phospho-CYLD (lane 3), suggesting phosphorylation of additional residues. The observed Ca2+/calmodulin-dependent mobility shift was blocked upon inclusion of the CaMKII inhibitor CN21 (lane 4) but phosphorylation at Ser-418 was, at least partially, preserved, suggesting phosphorylation of S-418 by additional kinases(s) present at the PSD. Two independent experiments yielded similar results. (TIF) [file pone.0091312.s002.tif]
