# Supplementary material for: CaMKII Mediates Recruitment and Activation of the Deubiquitinase CYLD at the Postsynaptic Density
Source: PLoS One. 2014 Mar 10;9(3):e91312. doi: 10.1371/journal.pone.0091312 (PMC3948843; doi:10.1371/journal.pone.0091312)
Supplement: Table S1 — Percent label intensity at the PSD compared to controls. In one EM experiment, sister hippocampal cultures were treated with either NMDA alone or NMDA plus APV (50 µM) for 2 min and labeled for either CYLD or CaMKII, followed by the quantification of immubolabeling intensities at the PSD. The differences between NMDA and NMDA+APV groups were statistically significant for both proteins (P<0.005, ANOVA with Tukey post-test). (DOCX) [file pone.0091312.s003.docx]

**Table**

**Table S1: Percent label intensity at the PSD compared to controls**

|  | **NMDA** | **NMDA+APV** |
| --- | --- | --- |
| **CYLD** | **248%** | **85%** |
| **CaMKII** | **262%** | **97%** |

In one EM experiment, sister hippocampal cultures were treated with either NMDA alone or NMDA plus APV (50µM) for 2min and labeled for either CYLD or CaMKII, followed by the quantification of immubolabeling intensities at the PSD. The differences between NMDA and NMDA+APV groups were statistically significant for both proteins (*P*<0.005, ANOVA with Tukey post-test).
